# Supernumerary Heterotopic Hemicerebellum: A Rare Case of an Accessory Mass in the Posterior Fossa

**DOI:** 10.5334/jbsr.2900

**Published:** 2022-10-11

**Authors:** Bert Verberckmoes, Caroline Vande Walle, Nele Herregods

**Affiliations:** 1 UZ Ghent, BE

**Keywords:** brain MRI, congenital malformations, posterior fossa malformations, hindbrain, pontine-medullar boundary, supernumerary heterotopic hemicerebellum

## Abstract

**Teaching Point:** A supernumerary heterotopic hemicerebellum is a rare congenital posterior fossa abnormality that should not be confused with tumor.

## Case History

A two-day-old boy was sent for an MRI of the brain in the neonatal work-up of a ‘cerebellar mass’ that was incidentally detected on prenatal ultrasound screening. To anticipate early intervention, he was born by elective caesarian section at 38 weeks. The brain MRI showed a probably benign exophytic seminodular hindbrain mass and along with the absence of postnatal complications, the intervention was called off. As expected, the mass showed no evolution on follow-up MRI performed six months later. Further clinical examination after birth remained normal.

**Figure 1:**
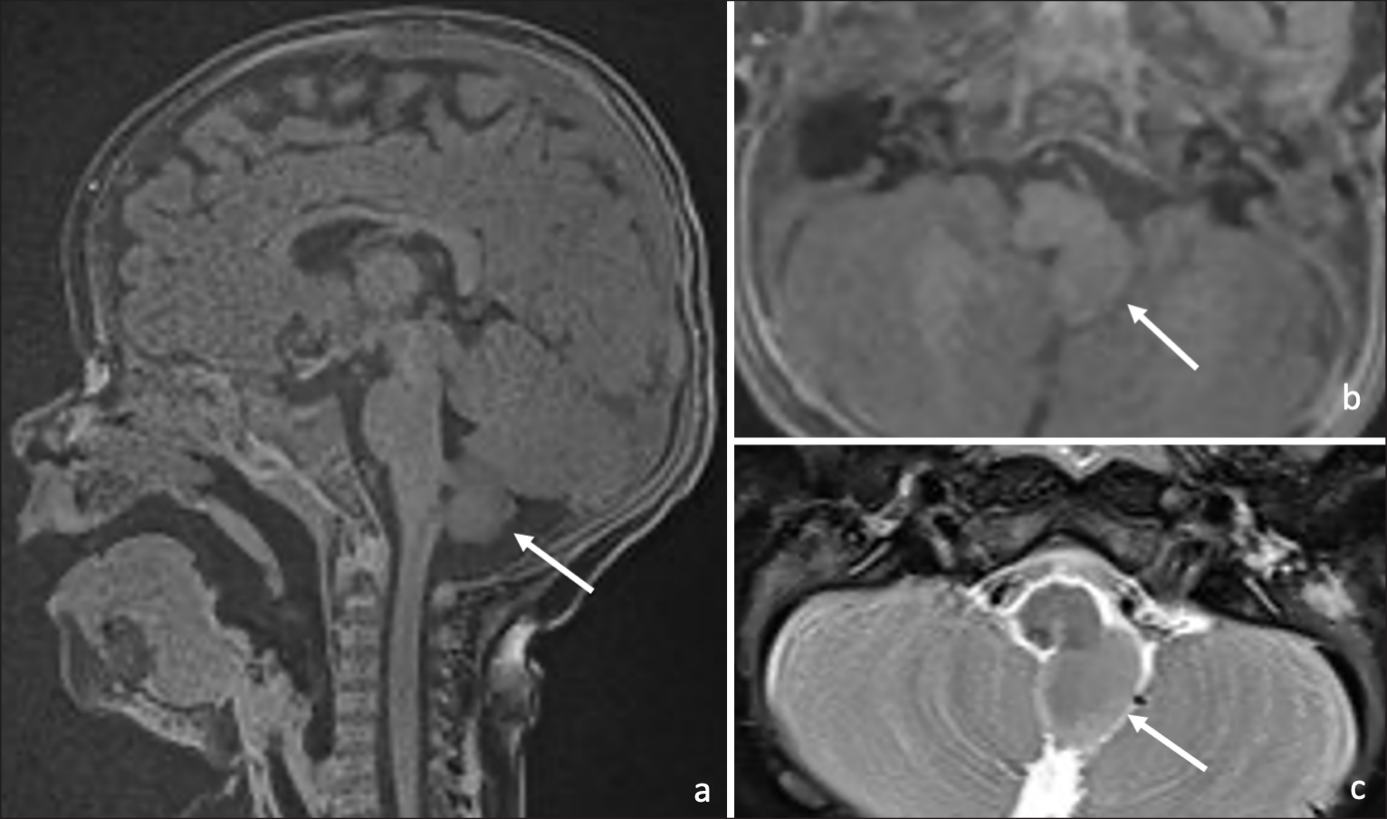


The exophytic seminodular hindbrain mass originated dorsally from the medulla oblongata on the left side. Superior, inferior, and lateral dysplastic soft tissue bridges were present between the mass and the dorsal medulla oblongata (Figure 1: [a] sagittal 3D fat-suppressed T1-weighted image with [b] axial reconstruction and [c] axial fat-suppressed T2-weighted spin echo image showing an exophytic semi-nodular soft tissue structure originating dorsally from the medulla oblongata on the left side (arrows)).

**Figure 2:**
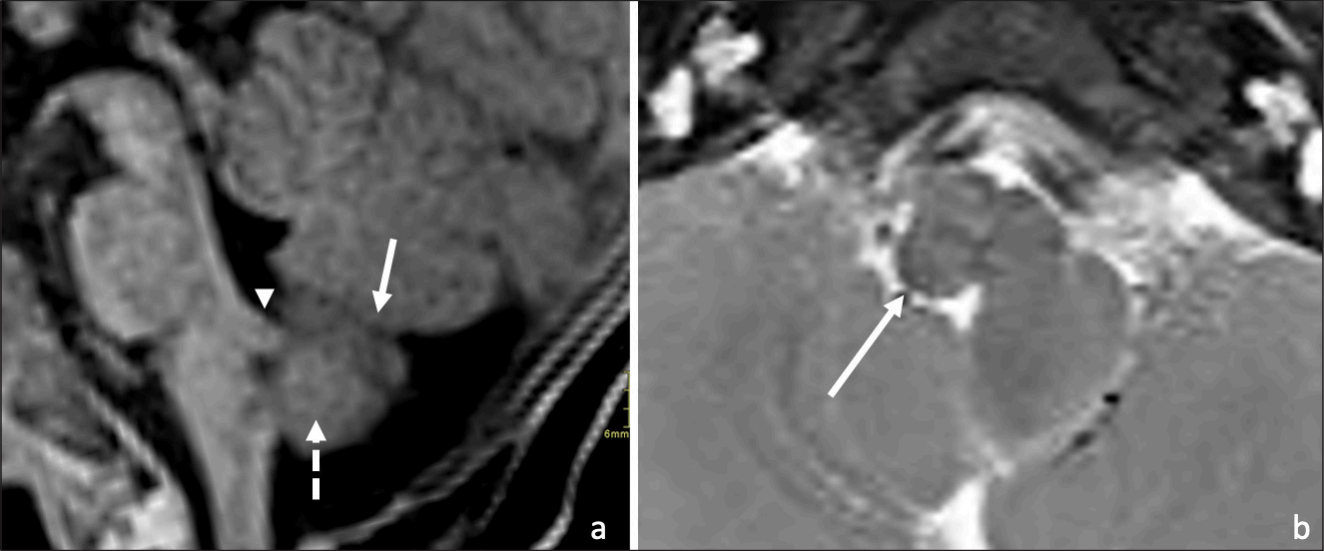


On both T1- and T2-weighted images, the signal intensity of the lesion was identical to that of the adjacent cerebellum, with white matter signal intensity in the center and cortical signal intensity in the periphery of the lesion. Mainly on the T1-weighted imaging, discrete foliation was noted. The lesion showed no diffusion restriction or enhancement after IV gadolinium administration (Figure 2: [a] sagittal 3D fat-suppressed T1-weighted image showing the central white matter intensity (dashed arrow) and peripheral cerebellar foliation (arrow) of the seminodular mass, also note the presence of a dysplastic superior soft tissue bridge (arrowhead); [b] axial fat-suppressed T2-weighted spin echo image showing a normal medulla oblongata on the right side (arrow)).

## Comment

Congenital midbrain-hindbrain malformations can roughly be separated into four major groups, according to Barkovich et al. in 2009, namely malformations caused by disturbances in the anteroposterior or dorsoventral ‘patterning’ of midbrain-hindbrain development, malformations associated with generalized neurodevelopmental disorders, localized brain malformations affecting the midbrain and hindbrain and combined hypoplasia and atrophy caused by putative prenatal neurodegenerative disorders.

A supernumerary heterotopic hemicerebellum is a very rare malformation. It is probably caused by patterning disturbances and cell fate transformations at the pontine-medullar boundary of the early hindbrain where a primitive supernumerary heterotopic hemicerebellum is induced on the alar plate of the medulla oblongata. This malformation can be included in group 1 of the midbrain-hindbrain malformations classification according to Barkovich.

A supernumerary heterotopic hemicerebellum as such has only been described earlier by Hattapoglu et al. in 2014. Other presentations of patterning disturbances in the posterior fossa have also been reported [1]. In some cases, an association is made with the PHACE syndrome.
